# Validation of an automated mite counter for *Dermanyssus gallinae* in experimental laying hen cages

**DOI:** 10.1007/s10493-015-9923-2

**Published:** 2015-05-23

**Authors:** Monique F. Mul, Johan W. van Riel, Bastiaan G. Meerburg, Marcel Dicke, David R. George, Peter W. G. Groot Koerkamp

**Affiliations:** Wageningen UR Livestock Research, P.O. Box 338, 6700 AH Wageningen, The Netherlands; Laboratory of Entomology, Wageningen University, P.O. Box 16, 6700 AA Wageningen, The Netherlands; Faculty of Health and Life Sciences, Northumbria University, Newcastle upon Tyne, NE1 8ST UK; Stockbridge Technology Centre, North Yorkshire, YO8 3TZ UK; Farm Technology Group, Wageningen University, P.O. Box 16, 6700 AA Wageningen, The Netherlands

**Keywords:** Integrated pest management (IPM), Automated counter, Validation, *Dermanyssus gallinae*, Poultry red mite, Laying hen

## Abstract

For integrated pest management (IPM) programs to be maximally effective, monitoring of the growth and decline of the pest populations is essential. Here, we present the validation results of a new automated monitoring device for the poultry red mite (*Dermanyssus gallinae*), a serious pest in laying hen facilities world-wide. This monitoring device (called an “automated mite counter”) was validated in experimental laying hen cages with live birds and a growing population of *D. gallinae*. This validation study resulted in 17 data points of ‘number of mites counted’ by the automated mite counter and the ‘number of mites present’ in the experimental laying hen cages. The study demonstrated that the automated mite counter was able to track the *D. gallinae* population effectively. A wider evaluation showed that this automated mite counter can become a useful tool in IPM of *D. gallinae* in laying hen facilities.

**Electronic supplementary material:**

The online version of this article (doi:10.1007/s10493-015-9923-2) contains supplementary material, which is available to authorized users.

## Introduction

Integrated pest management (IPM) is a method that is frequently used in numerous cropping systems. This method is based on the integration of all ecological and biological knowledge about a certain pest species, including the effect of both biotic and abiotic factors on population development . IPM aims to minimize economic losses by including different environmentally safe methods to prevent and control pests whilst deploying pesticides only as a last resort, thus reducing issues with pesticide contamination and resistance (Anonymous 1969). With IPM, an action threshold is typically set and the pest species is closely monitored so that any kind of treatment is only deployed as necessary, and when any preventative measures have failed. If monitoring indicates that the economic threshold of the pest population is exceeded, effective interventions (e.g. extra interim releases of biological controls or application of pesticides) are initiated. Consequently, IPM requires efficient pest monitoring for optimal deployment of the interventions used. In livestock production systems, application of all but basic IPM programmes is still relatively rare. This is despite the fact that livestock systems could benefit from application of this approach (e.g. by reduced economic losses), poultry egg production included (Sparagano et al. 2014).

The poultry red mite (*Dermanyssus gallinae* De Geer) is a significant pest of egg laying hens, present in a large percentage of layer houses worldwide (Sparagano et al. 2014). *Dermanyssus gallinae* is generally referred to as an ectoparasitic mite, though based on its feeding behaviour it is perhaps better described as a micropredator (Lafferty and Kuris 2002). *Dermanyssus gallinae* feeds on the blood of numerous avian hosts, including laying hens, though they may also pose a risk to poultry workers (George et al. 2013). *Dermanyssus gallinae* requires a blood meal for development from protonymph to deutonymph, and from here to the adult stage (Axtell and Arends 1990), with feeding also being required for adult female reproduction. Mite feeding upon hens causes agitation of the birds, and where pest populations proliferate they may even result in anaemia (Sikes and Chamberlain 1954; Kilpinen et al. 2005). High economic losses are associated with *D. gallinae* infestations, with costs of pest control and production losses estimated at more than 130 million euro per year for the EU egg industry (Emous et al. 2005). Even at low population levels, *D. gallinae* pose a risk of disease transmission within the flock, being implicated as vector for numerous poultry pathogens (Sparagano et al. 2014).

Control of *D. gallinae* is difficult due to the fact that this species spends the majority of its time secluded in hard-to-target refugia within the sub-structure of the poultry unit. Mites aggregate off-host in cracks and crevices where they seek shelter to digest their blood meal, where protonymphs and deutonymphs molt, and where adults mate and lay eggs. They only emerge, preferably during darkness, to feed and spend just 30–60 min on the hen during an average visit (Maurer et al. 1988). This hampers successful treatment with standard acaricides that need to contact the target to have an effect. Currently authorized acaricidal products display shorter residual activities to satisfy lowered maximum residue limits (MRLs) than conventional acaricides, many of which have been withdrawn from the market. However, shorter residual efficacies are ill-suited to target *D. gallinae* which may not encounter treated surfaces for several days (or more) after application. Successful treatment is also hampered by the ability of *D. gallinae* to develop resistance to multiple acaricides (Chauve 1998; Nordenfors et al. 2001; Marangi et al. 2009).

In order to achieve better control of *D. gallinae* in laying systems, several authors have proposed a more rigorous implementation of IPM, which is currently largely limited to some combination of biosecurity, acaricide use and clean down between flocks (Arends and Robertson 1986; Harrington et al. 2011; Sparagano et al. 2014). Monitoring is a key factor in facilitating the development of IPM regimes for *D. gallinae* (Sparagano et al. 2014) and a number of relatively basic monitoring methods are available (Table 1).

**Table 1:** Description of the most frequently used methods for monitoring *Dermanyssus gallinae*

Monitoring method	Limitations	Reference
1. ADAS© Mite Monitor	Labour intensive; not sensitive to very small populations	Anonymous (2014)
2. Perch trap	Labour intensive; not easily applicable in most poultry facilities	Kirkwood (1963)
3. Tube containing a fabric or cloth	Labour intensive; not sensitive to very small populations	Maurer et al. (1993)
4. Corrugated cardboard/plastic trap	Labour intensive	Nordenfors et al. (1999)
5. A tube trap with a wooden stick or corrugated cardboard	Labour intensive; indicates trends only (infestation rates from 0–4); not sensitive to very small populations	van Emous and ten Napel (2007)
6. Detecting *D. gallinae* in dust, feathers and impurities	Labour intensive; not sensitive to very small populations; sub-optimal sampling site specification	Pavlicevic et al. (2007)
7. Examining dried droppings for presence of *D. gallinae*	Labour intensive; not sensitive to very small populations; sub-optimal sampling site specification	Zenner et al. (2009)
8. Mite monitoring Score (MMS) method	Labour intensive; indicates trends only (infestation rates 0–4); not sensitive to very small populations	Cox et al. (2009)

Generally, the major disadvantages of existing mite monitoring methods is that they are labour intensive and only give a rough indication of population growth or decline in mite populations (Mul et al. 2009). As a result existing mite monitoring methods are scarcely used by egg producers. Where monitoring is undertaken, this is typically achieved using ‘traditional’ methods (1–5 in the Table 1), involving the use of passive and static refuge traps (e.g. corrugated card board). Unless carefully positioned at multiple sites, with consideration given to mite aggregation and feeding behaviour, such monitoring methods can easily underestimate *D. gallinae* infestation levels.

As a solution to the monitoring problems reported above we have previously designed, developed and tested an automated mite counter (Mul and Ploegaert 2014).This counter was deliberately designed to monitor a *D. gallinae* population in a layer house in an economically feasible manner using low cost but durable materials and solutions. The aim of the current study was to assess the validity of this counter to monitor a range of *D. gallinae* population sizes from small (when only a few specimens are present and infestations are visually undetectable) to large (when clusters of *D. gallinae* are visible). Here we present the outcome of this validation study under semi-controlled conditions, and evaluate the contribution of this automated mite counter to a more effective IPM regime in laying hen facilities.

## Materials and methods

Experimental laying hen cages, containing live birds and one automated mite counter, were repeatedly experimentally infested with a known population of *D. gallinae*. During defined time periods, initial mite populations were supplemented to established populations to achieve five levels of infestation (after Cox et al. 2009 see Table 2). The automated mite counter was validated by comparing the data returned by the automated counter (# of mites counted) to the absolute counts of *D. gallinae* present in the cages (# of mites present).

**Table 2:** Classification of *Dermanyssus gallinae* population levels (see Cox et al. 2009)

Level	Characteristics
0	No mites visible
I	Mites visible in cracks and crevices
II	Mites visible at unprotected places
III	Clusters of mites (groups of mites larger than 1 cm^2^) visible in cracks and crevices
IV	Clusters of mites (groups of mites larger than 1 cm^2^) visible at unprotected places in and on the experimental cages

### Mites in the experiment

During the experiment, mites originating from one of the two different Dutch layer farms used as sources of *D. gallinae* were released in the experimental cages in predefined numbers. These mites were collected in the morning and only viable nymphs and adults, that were able to walk on a petri dish, were used for the experiment and placed in a plastic vial with screw cap (102 mm height, 52 mm diameter, VWR International BV). At the end of the afternoon, in the dark, the vials were placed under the laying nests of the hens in the experimental cages and the screw caps were removed.

To ensure that comparable populations of *D. gallinae* were used throughout the study, the longevity of the mites from both farms in the laboratory was compared by determining the number of surviving mites after 14 days (n = 6). A Student’s *t* test confirmed that the longevity of mites from the two farms was similar (*P* = 0.35).

### Layout of experimental laying hen cages

**Fig. 1:**
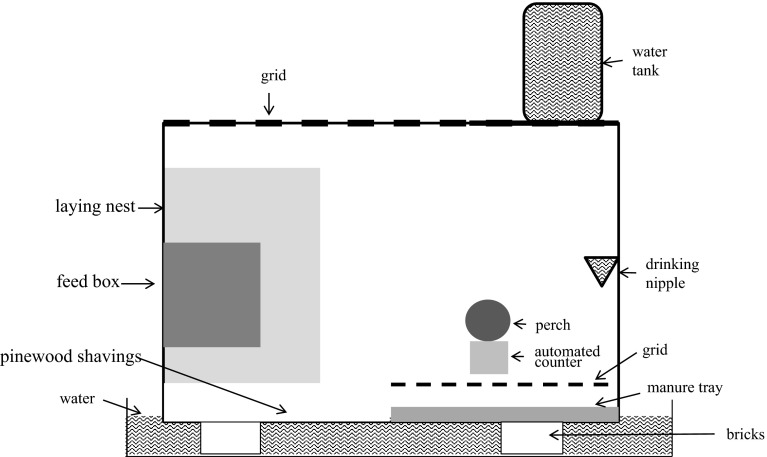
Schematic cross-section of the experimental laying hen cage

Twelve experimental laying hen cages (Fig. 1) were used during the experiment. These cages were confirmed as being free of *D. gallinae* prior to the start of the experiment by (1) thorough cleaning, (2) visual inspection and (3) confirmation by zero counts of the automated counter during 2 days prior to the start of the trial. Cages (1.2 × 0.6 × 0.6 m) were open at the top, but to prevent the hens from escaping a metal grid with an access hole was used as a cover. The front of the cages was made of a transparent Perspex plate, and the structure of each cage was supported by two wooden beams. In each cage, approximately 50 % of the floor surface was filled with pine wood shavings. A manure tray, covered with a grid, filled the remaining floor surface. Above this manure tray, a metal perch was fixed through the front and rear cage wall. Under the perch, the automated mite counter was placed. Above the litter area, a feed box and a laying nest (0.3 × 0.4 × 0.5 m with a hole of 0.2 × 0.3 m) were placed. A drinking nipple with leakage cup was also placed above the manure tray, close to the right-hand wall. The nipple was connected to a closed water tank placed on a transparent plate on top of the metal grid. The whole cage was placed on bricks in a tray filled with water to ensure that mites could not move between different cages.

Each cage housed two ‘Specific Pathogen Free’, mite-free, beak-trimmed laying hens being 29 weeks of age at the start of the experiment (white leghorn, GD Animal Health, The Netherlands). Hens had never been exposed to acaricides nor treated against worms. Hens were assigned to cages based on their weight and allowed to settle for 2 days before mites were added. Feed was provided *ad libitum* with a commercial layer feed crumble, stored at −20 °C prior to use. Water was constantly available and a 16:8 light:dark light regime was implemented, switching the lights off at 06:00 p.m. Hen health and welfare were checked daily by employees of the experimental farm of the Central Veterinary Institute of Wageningen UR, according to all legal requirements set by Dutch law (Approved Experimental Number 2013145).

### Automated mite counter

**Fig. 2:**
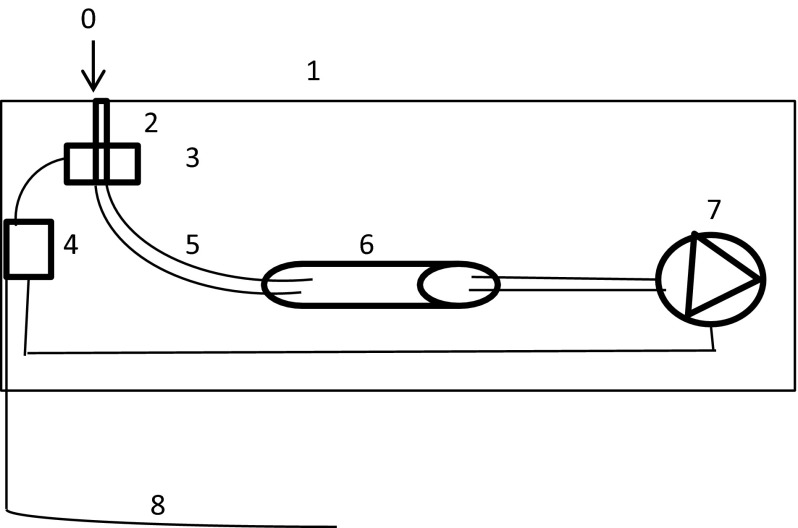
Schematic cross-sectional view of the automated mite counter, including an opening (diameter approximately 1–1.5 mm) to the tube (0) in a body (1), comprising a casing and a lid closing the casing, a receiving section, (2) a sensor device for counting the passing mites (3), an electronic processor (4), a tube (5), filter (6), a removal device using air suction (7) and a power and data cable (8)

Within each cage, an automated mite counter (Fig. 2) was fixed to the perch using cable ties and tape. The entrance of the counter (0) was positioned in the lid of the case next to the bottom of the round metal perch (Fig. 1). Mites entering the counter through a hole (diameter of 1–1.5 mm) in the lid (1) were detected by a sensor device (3) when passing the sensor. When a mite was detected, the sensor device passed a signal to the processor (4), which subsequently switched on the insect removal device (7) to remove the mites in front of the sensor, by air suction, into a filter (6). This filter contained the mites until it was emptied outside the cage, at weekly intervals, to prevent blockage of the air flow. We assumed it very unlikely that the mites were able to escape from the filter as this would have required overcoming a series of physical obstacles. This was confirmed by the fact that no mites were counted during light periods. We assumed that, since it was dark on the inside of the counter, mites would have been willing to move within the counter at any time of the day.

Prior to the experiment, the sensitivity of the automated counter was assessed *in vitro*, revealing that it was able to detect 100 % of adult *D. gallinae* and 97 % of nymphs and larvae (n = 35 per life stage). All automated counters used in the experiment were also checked in vitro using both live mites and dummies (i.e. thin electrical wire of 0.09 mm diameter) and adjusted until no failure was detected and the sensor was registering the wire and live mites (all stages) with the pump activated and stopped properly.

During the experiment, every 5 min the counted number of mites in each cage were registered and saved by an external data logger that was connected with the automated counter by a cable (8), starting from 2 days before the first mite release (to ensure zero counts) until the end of the study. For analysis, the number of mites counted by the automated counter (‘# of mites counted’), was the summed total of all counted mites starting from 10.00 a.m. (after caretaking activities) on the day prior to mite collection until 08.00 a.m. on the day of mite collection.

### Collection and counting of mites from cages

In order to obtain absolute counts of *D. gallinae* present in the cages (‘# of mites present’), mites in and on the following materials were collected separately: (a) wet manure (from the manure tray); (b) dry part of the manure and litter (in the manure tray, two types of manure were found; the dry part where the manure is mixed with litter, and wet manure without litter); (c) dried manure attached to the grid above the manure tray; (d) dust in the laying nest; (e) surfaces of the cage (in- and outside); (f) surfaces of grid and transparent plate on top of the cage; g) perch; (h) outside of the laying nest and feed box; (i) water tank; and (j) wooden supporting beams. For a–d, manure and dust were collected into large transparent plastic bags and the number of mites present was determined by a two-step process. Initially, mites that aggregated at the top of the whole sample bag after 24 h (dust) or 48 h (manure) were counted and removed. Following this, four sub-samples of 1–2 g were removed after mixing and checked for mites under a microscope (variable magnification of 0–23×). The total number of mites present in whole samples was calculated by multiplying up the number of mites in the sub samples (i.e. by multiplying by 100/weight percentage of the sub sample), and adding the number of aggregated mites at the top of the whole sample bag. For e–j, samples were collected by brushing the mites from the surfaces with a broad brush (Elma 59-1 type) into a large glass vial (0.2 m diameter × 0.09 m height) containing 70 % alcohol, from which mites were then counted. Low numbers of mites in the alcohol were counted in full under the microscope. Higher numbers of mites in the alcohol were sub-sampled. After stirring for a homogeneous distribution of mites in the alcohol layer, 10 % of the sample was placed in petri dishes (0.14 m diameter). The mites present in two sections (equivalent to 1/8 of the solution) were then counted using a binocular microscope. The number of mites present per cage (‘# of mites present’) at the day of collecting the mites was calculated as the sum from all counted and calculated mites present in all samples (a–j) per cage and the number of mites found on the hens per cage at the day of collecting the mites.

The mites counted by the automated mite counter were collected in the filter and remained there until the filter was refreshed. Mites in filters were not visually counted, nor compared with the number of mites counted during the monitoring period, because the mites (1) were able to produce eggs, larvae and protonymphs, and (2) were often crushed at high population levels, thus making it impossible to distinguish individual mites.

### Set up of the experiment

The experiment was conducted in three phases; A, B and C. To achieve *D. gallinae* population sizes representing the five levels of Cox et al. (2009), low and high mite infestations treatments were released in the cages with a ratio of 1:10; low infestation treatment: high infestation treatment. Half of the mites released in the cages were adults (mixed gender) and the remaining half were nymphs. Activities undertaken during the experiment are summarized in Table 3.

**Table 3:** Activities per day during the validation experiment of 75 days split up in three phases A, B and C

Phase	Day	Activity
A	0	Release of mites. Low infestation (LI) cages: 50; 25 nymphs and 25 adults High infestation (HI) cages: 500; 250 nymphs and 250 adults
	7	Filters refreshed
	14	Manure tray emptied Filters refreshed
	28	Collecting and counting of mites in two HI cages and two LI cages, mites on the hens counted and hens were culled (cages taken out of experiment)
	33	Collecting and counting of mites in two HI cages, mites on the hens were counted, the hens were not culled
		All eight remaining cages were emptied, cleaned and dried
B	33	All hens and automated counters returned to their own cages
	34	Release of mites LI cages: 250; 125 nymphs and 125 adults HI cages: 2500; 1250 nymphs and 1250 adults
	38	Release of mites LI cages: 250; 125 nymphs and 125 adults HI cages: 2500; 1250 nymphs and 1250 adults
	40	Filters refreshed
	44	Release of mites LI cages: 250; 125 nymphs and 125 adults HI cages: 2500; 1250 nymphs and 1250 adults
	47	Filters refreshed Manure tray emptied
	50	Release of mites LI cages: 250; 125 nymphs and 125 adults HI cages: 2500; 1250 nymphs and 1250 adults
	54	Collecting and counting of mites in four HI cages and two LI cages, mites on the hens were counted, the hens were not culled
		All eight cages were emptied, cleaned and dried
C	54	All hens and automated counters returned to their own cages
	55	Release of mites LI cages: 500; 250 nymphs and 250 adults HI cages: 5000; 2500 nymphs and 2500 adults
	58	Release of mites LI cages: 500; 250 nymphs and 250 adults HI cages: 5000; 2500 nymphs and 2500 adults
	61	Filters refreshed
	68	Manure tray emptied Filters refreshed Release of mites LI cages: 500; 250 nymphs and 250 adults HI cages: 5000; 2500 nymphs and 2500 adults
	72	Release of mites LI cages: 500; 250 nymphs and 250 adults HI cages: 5000; 2500 nymphs and 2500 adults
	75	Collecting and counting of mites in four HI cages and two LI cages, mites on the hens counted, all hens were culled
		End of experiment

#### Phase A, Day 0–33

In Phase A twelve cages were randomly allocated to treatments: six cages to a low-infestation treatment group (50 mites; 25 nymphs and 25 adults) and six cages to a high-infestation treatment group (500 mites; 250 nymphs and 250 adults).

The relative humidity in the experimental unit was set at 70 ± 5 % and the temperature was set at 27 ± 5 °C. The manure tray was emptied on day 14 (post mite release), leaving the dry manure near the litter area in situ. The filters of the automated counters were refreshed on days 7 and 14.

Twenty-eight days after releasing the mites, two cages from the low infestation treatment group and two cages from the high infestation treatment group were removed from the study and the mites in the cages were collected and counted. Mites present on the hens from these cages were also counted. Thirty-three days after mite release counts were made from a further two cages from the high infestation treatment group. Mites present on the hens from these two cages were again counted at this time. These last two cages were not removed from the study, but used in Phases B and C.

#### Phase B, Day 33–54

At the start of Phase B, hens were removed from the eight remaining cages and these cages were subsequently cleaned thoroughly with water and chlorine before drying with paper. The automated mite counters and the hens were then returned to these cages.

The relative humidity of the experimental unit was lowered to 60 ± 5 % and the temperature was set at 25 ± 5 °C to limit fungal growth (as observed in Phase A). On days 34, 38, 44 and 50, mites were released in four cages of the low infestation treatment group (250 mites per release and per cage; 125 adults: 125 nymphs) and in four cages of the high infestation treatment group (2500 mites per release and per cage; 1250 adults: 1250 nymphs). On the morning of day 47, the manure tray was emptied, leaving the dry manure near the litter area in situ. The filters of the automated mite counters were refreshed on days 40 and 47. On day 54 all of the mites from six of the eight cages were collected; two cages from the low-infestation treatment group and four cages from the high-infestation treatment group. Mites could not be collected from the remaining two cages due to limited availability of labour.

#### Phase C, Day 54–75

At the start of Phase C, all eight cages used in Phase B were cleaned and restocked as previously described. On days 55, 58, 68 and 72, mites were released in four cages of the low infestation treatment group (500 mites per release and per cage; 250 adults: 250 nymphs) and in four cages of the high infestation treatment group (5000 mites per release and per cage; 2500 adults: 2500 nymphs). On the morning of day 68, the manure tray was emptied, leaving the dry manure near the litter area in situ. The filters of the automated mite counters were refreshed on days 61 and 68. On day 75 all of the mites from six of the eight cages were collected; two cages from the low-infestation treatment group and four cages from the high-infestation treatment group. Mites could not be collected from the remaining two cages due to limited availability of labour.

### Statistical analysis

An analysis was performed to assess the degree to which ‘# of mites counted’ corresponded with ‘# of mites present’. Linear regression analysis was performed within a restricted maximum likelihood (REML) variance component analysis using the GenStat software (16th edition) (Anonymous 2006). Parameters were estimated by REML (Searle et al. 1992). Both variables (‘# of mites counted’ as the dependent variable and ‘# of mites present’ as the regression variable) were ln transformed. Differences between “cages” were estimated by a random effect ε_i_.

The line of regression wherein the relationship was modelled between the ‘# of mites counted’ and the ‘# of mites present’ was as:

$$ {\text{Ln}}\left( {{\text{Y}}_{{{\text{ij }}({\text{mites}}\, {\text{counted}})}} } \right) = \,\upbeta_{0} + \,\upbeta_{1} *\ln \left( {{\text{Y}}_{{{\text{ij }}({\text{mites}} \, {\text{present}})}} } \right) \, + \underline{\upvarepsilon}_{\text{i}} + \underline{\upvarepsilon}_{\text{ij}},$$

with: Y_mites counted_ = number of counted mites by the automated counter during the last 22 h; β_0_ = intercept; β_1_ = regression coefficient; Y_mites present_ = reference = number of mites in and on cage i (1–9) of phase j (A,B,C); $\underline{\varepsilon }_{\text{i}}$ = random effect of cage i with N(0, $\sigma_{\text{i}}^{2}$); $\underline{\varepsilon }_{\text{ij}}$ = residual effect of cage i, phase j with N(0, $\sigma_{\text{ij}}^{2}$).

As ‘# of mites counted’ was analysed on ln scale, the relative standard deviation (S_r_) was calculated by taking the square root of the residual variance (${=}S_{ij}^{2}$). This S_r_ was multiplied by a Student’s *t* value to yield a two-sided confidence interval for individual measurements.

$$ 95\,\% - CI_{r} = S_{r} *t_{(0.975;\,n - 2) }$$

## Results

The cages removed in Phase A showed very low numbers of mites present, possibly due to heavy infestation with fungi (*Aspergillus* spp., *Penecilium* spp., *Mucor* spp.). Therefore all remaining cages were thoroughly cleaned before commencing Phase B and C. Nevertheless, three data points of ‘# of mites counted’ and ‘# of mites present’ obtained from Phase A were included in the dataset. Seventeen data points of ‘# of mites counted’ and ‘# of mites present’ were obtained for analysis; across all phases of the experiment, single sets of counts (‘# of mites counted’ and ‘# of mites present’) were taken from three cages, two sets of counts were taken from four cages, and three sets of counts were taken from two cages. These data points were obtained from nine out of twelve available cages. One cage from Phase A was excluded since a zero mite count was returned by the automated counter and zero mites were present in that cage. From Phase B and Phase C, two cages with low mite infestations were excluded since the ‘# of mites present’ were never determined due to the limited availability of labor.

The intercept (β_0_) and the regression coefficient (β_1_) were determined as −1.25 (95 % confidence interval: −2.64, 0.14) and 0.67 (95 % confidence interval: 0.50, 0.84), respectively.

**Fig. 3:**
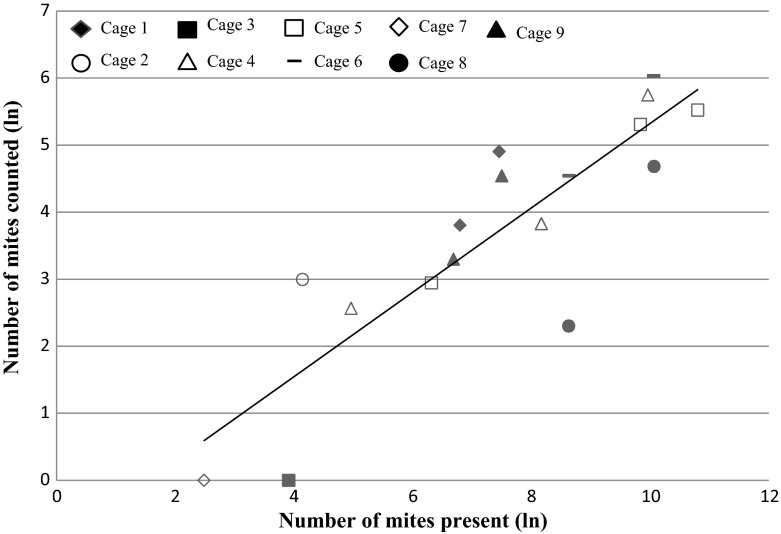
Measured data points for cage 1–9 and modelled relationship (*line*) for the number of mites present in the cages versus the number of mites counted (both ln transformed)

The 17 data points and the modelled regression line obtained from the analysis are shown in Fig. 3. The Goodness of fit (R-square) of this line was 90.3 %, supporting that about 90 % of the variation in ‘# of mites counted’ was explained by the level of the reference value (‘# of mites present’). The value for 95 %-*CI*_*r*_ was 1.17, meaning that 95 % of the individual measurements were in the relative range of (−117 %; +117 %).

The slope of the regression (β_1_) was smaller than 1, meaning that there is a relationship between the number of mites present and the number of mites counted. When there is a relative increase in number of mites present, however, the increase in the number of mites counted was not increased by the same percentage, but rather by a factor 1.6 (i.e. with higher mite populations present in the cages a lower percentage of the population was detected). Ergo, it appears that at higher mite densities the counter is increasingly conservative. Nevertheless, relative increases in the number of mites present did correspond to an increase in the number of counted mites, thus demonstrating that the automated mite counter is able to track and detect a mite population increase.

It should be noted that there was a significant effect of cage (*P* < 0.05). The counters “1” and “8” deviated more from the predicted line than the other counters. The counter in cage “1” counted significantly more mites (*P* < 0.001) compared to the average of the other counters. The counter in cage 8 counted significantly fewer mites (*P* < 0.01) compared to the average of the other counters. These systematic effects were included in the random term $\underline{\varepsilon }_{\text{i}}$.

## Discussion

The aim of this study was to validate a recently-developed automated mite counter for *D. gallinae* to determine its ability to monitor a range of *D. gallinae* population sizes. A range of experimental mite populations, from small to large, were achieved in populated cages to meet this aim, covering all five levels of *D. gallinae* infestation according to Cox et al. (2009); 0 = no mites visible; I = Mites visible in cracks and crevices; II = Mites visible at unprotected places; III = Clusters of mites (groups of mites larger than 1 cm^2^) visible in cracks and crevices; IV = Clusters of mites (groups of mites larger than 1 cm^2^ visible at unprotected places in and on the experimental cages).

The results demonstrated that the automated mite counter was able to track and detect *D. gallinae* population growth. When mite populations increased, so did the number of mites counted. Moreover, when a decline of the mite population was visually observed during the study, the number of mites counted by the automated mite counter also decreased (personal observation MF Mul).

A strong correlation was observed on the ln-ln scale between the ‘# of mites counted’ and the ‘# of mites present’, with an estimated intercept (β_0_) of −1.25. Although the intercept in the model was not strongly significant (*P* < 0.10), it was included in the model to avoid the regression line on a ln-ln scale passing through the zero points of the axes. On the original scale such a regression line would intersect at (1,1), which we assumed not to be realistic.

**Fig. 4:**
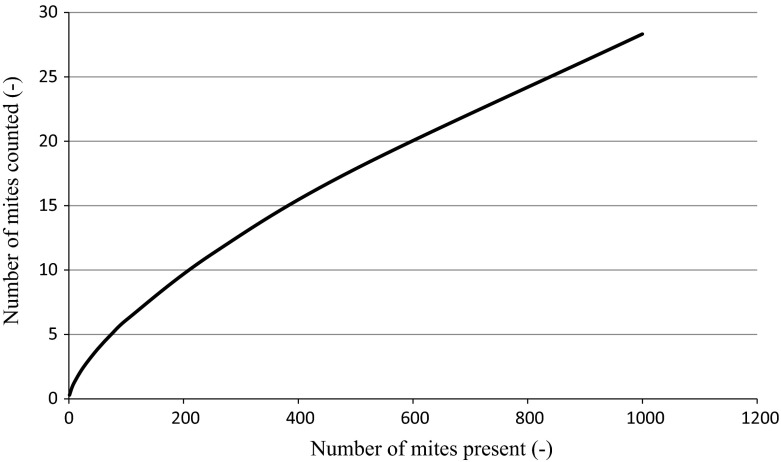
Line showing the regression between the number of mites present in the cages and the number of mites counted (real scale)

The regression coefficient (β_1_) of the regression line was 0.67 in the current study, meaning that an increasing number of mites present in the cages resulted in a slightly smaller increase in the number of counted mites (as shown in Fig. 4 on real scale). One possible explanation for this could be that the presence of abundant fungi in Phase A may have impaired the mites, or even killed them, as suggested by Tavassoli et al. (2011) and supported by personal observation in the current study (MF Mul). Post feeding, relatively low populations of impaired mites in Phase A may have been more responsive to the shelter provided by automated counters, with these mites being less able to travel greater distances to locate other refugia than ‘healthier’ mites present in other Phases.

Another possible explanation for the fact that an increasing number of mites present in the cages resulted in a relatively smaller increase in the number of counted mites could be the presence of aggregation cues from conspecifics. Pheromones are known to be attractive to *D. gallinae* (Koenraadt and Dicke 2010) and at lower population levels directional responses to these may have been weaker, with mites more likely to ‘wander’ and encounter the monitor as a result. The thigmokinetic response of *D. gallinae* to conspecifics (Entrekin and Oliver 1982) might have similarly contributed to lower-than-expected counts at higher mite population levels, with mites more likely to be arrested by contact with conspecifics (and thus less likely to ‘wander’ and encounter monitors) at higher *D. gallinae* population levels. In support, at high infestation levels in the current study, we noticed aggregations of mites in and on the experimental cages that were not seen at lower levels.

Though counters were designed to be attractive to mites, providing a heat source (created by the internal processor) which should have attracted host-seeking mites (Kilpinen 2001), it is possible that pheromone cues provided a stronger stimulus (Koenraadt and Dicke 2010). This would be especially true for *D. gallinae* that had already fed, with Entrekin and Oliver (1982) observing that fed female mites cluster more rapidly than unfed females. As live hens may have provided a stronger heat stimulus (coupled with additional chemical stimuli) than counters, it is further possible that counters were more likely to record visits from fed *vs* unfed mites in the current study, further exacerbating any relatively reduced counts at higher population levels. With future development of our mite monitoring tool it may be possible to overcome this constraint, for example by including a chemical attractant within the counter. However, although research has shown that chicken odours are attractive to *D. gallinae*, and that these mites produce an aggregation pheromone (Koenraadt and Dicke 2010), no synthetic attractants are presently available for this species. Moreover, including attractants would increase the systems maintenance time as these would need to be refreshed. A simpler solution in the short term, at least until attractants are developed for *D. gallinae*, is to accept that the monitor counts relatively fewer mites at higher infestation levels and correct for this accordingly.

A strong requirement for a good monitoring tool to improve IPM for *D. gallinae* is the detection of low numbers of mites. Monitoring mite population growth at low infestation levels is necessary to inform timely management interventions and reduce the detrimental effects of *D. gallinae* infestations (Mul and Koenraadt 2009). The reclusive life-style of *D. gallinae*, however, makes it more difficult to identify low mite population levels. As shown in Fig. 3, the automated counter is able to provide a good impression of the number of mites present, even when the infestation is low. Although only three out of 17 data points used in the analysis had relatively low numbers of mites present in the cages (mites present ≤4; cage 2, 3 and 7 in Fig. 3), daily number of mites counted per cage support that the system is able to detect day-to-day variation and is sensitive to daily changes in the mite population from low to high levels (see Online Resource 1; Fig. 1 showing the daily counts of the automated mite counter during phase C as an example).

The value for 95 %-*CI*_*r*_ in the current work was 1.17, demonstrating a substantial error in the predicted number of mites counted at certain levels of mites present. Analysis revealed a significant cage effect (*P* < 0.05) which could explain this large confidence interval, but as counters and cages were coupled it is not possible to separate the two, and equally possible that variability in counters (or any combination of cages and counters) led to a relatively high 95 %-*CI*_*r*_. In future studies we aim to determine the measurement error of the counter, using multiple mite counters around one monitoring site, thereby allowing us to be able to reduce the prediction error. The need for multiple mite counters to monitor a *D. gallinae* infestation in poultry facilities is supported by Nordenfors and Höglund (2000), who recommend the use of multiple mite counters to compensate for the spatial differences in *D. gallinae* distribution.

In future commercial practice we envisage that the automated counter (and the statistical model) validated here will be effectively reversed for use: i.e. the counted number of mites will provide a prediction of the population at a certain point in time. The farmer will be informed on mite population development and effectiveness of treatment interventions. To this end, detecting relative changes of the population size over time will be more important than assessing the absolute number of mites at a given time-point to warn the egg producers about the growth or decline of the *D. gallinae* population. Though the current work focused on validating the counter based on single time-point counts, daily number of mites counted per cage (see Online Resource 1; Fig. 1) support that temporal changes can be detected by this system.

An IPM approach results in a more effective and economic control of pest species (Metcalf and Luckmann 1982). Monitoring the development of a pest population is a key factor of IPM as it indicates the moment that the action threshold of the pest population is exceeded. Monitoring also clearly shows the effect of a treatment or management measures, which are applied after any preventive measures have failed (Zehnder 2014). However, it is of utmost importance that the monitored pest is the same pest against which preventive measures are taken. The validated automated mite counter was developed for counting *D. gallinae* and was constructed based upon knowledge of the behavior of *D. gallinae*. When we were able to check all species in the filter during the experiment and the development of the automated counter in the layer house, we solely found *D. gallinae* in the filters, supporting that the automated mite counter only counts *D. gallinae* (pers. observation MF Mul).

After identifying the pest species, it is necessary to monitor the pest population, as with IPM no treatments are applied unless the pest is present and poses a threat (Zehnder 2014). Even though the requirements for a monitoring tool applicable for IPM are different per pest species, there are some general requirements. A monitoring tool applicable for IPM should be (1) able to detect small population sizes, (2) able to monitor a relative change of the population size in time, (3) able to monitor at relevant frequency intervals (for example with weekly intervals as the production data also is available on a weekly basis), (4) able to monitor the population throughout a facility as the spatial distribution of mites may change over time and, (5) carried out with a minimum of labor (Dively 2014). The tested automated mite counter meets all of these five requirements, especially when multiple mite counters are evenly spread throughout the poultry facility.

In conclusion, the automated mite counter developed and tested here is a potentially useful tool for the application of IPM against *D. gallinae* in layer production systems. It automatically monitors the mite population, even when the population is small, and meets all other requirements for an IPM monitoring tool as specified by Dively (2014). Using multiple mite counters in a closed environment (e.g. laying hen facility), monitoring the pest population size and spatial-temporal growth will be relatively straight-forward and less labour intensive when compared to other available *D. gallinae* monitoring methods (see Table 1). Coupled with the development of an operational population model for *D. gallinae* and determination of an economic threshold, this system could promote comprehensive IPM regimes for *D. gallinae* (Sparagano et al. 2014) resulting in improved detection and control of this pest. The next step for the system is to pursue commercial development, and this is currently underway.

## Electronic supplementary material

(PPTX 77 kb)
