# Supplementary material for: Validation of an automated mite counter for Dermanyssus gallinae in experimental laying hen cages
Source: Exp Appl Acarol. 2015 May 23;66(4):589–603. doi: 10.1007/s10493-015-9923-2 (PMC4481303; doi:10.1007/s10493-015-9923-2)
Supplement: Supplementary file 1 — (PPTX 77 kb) [file 10493_2015_9923_MOESM1_ESM.pptx]

## Slide 1
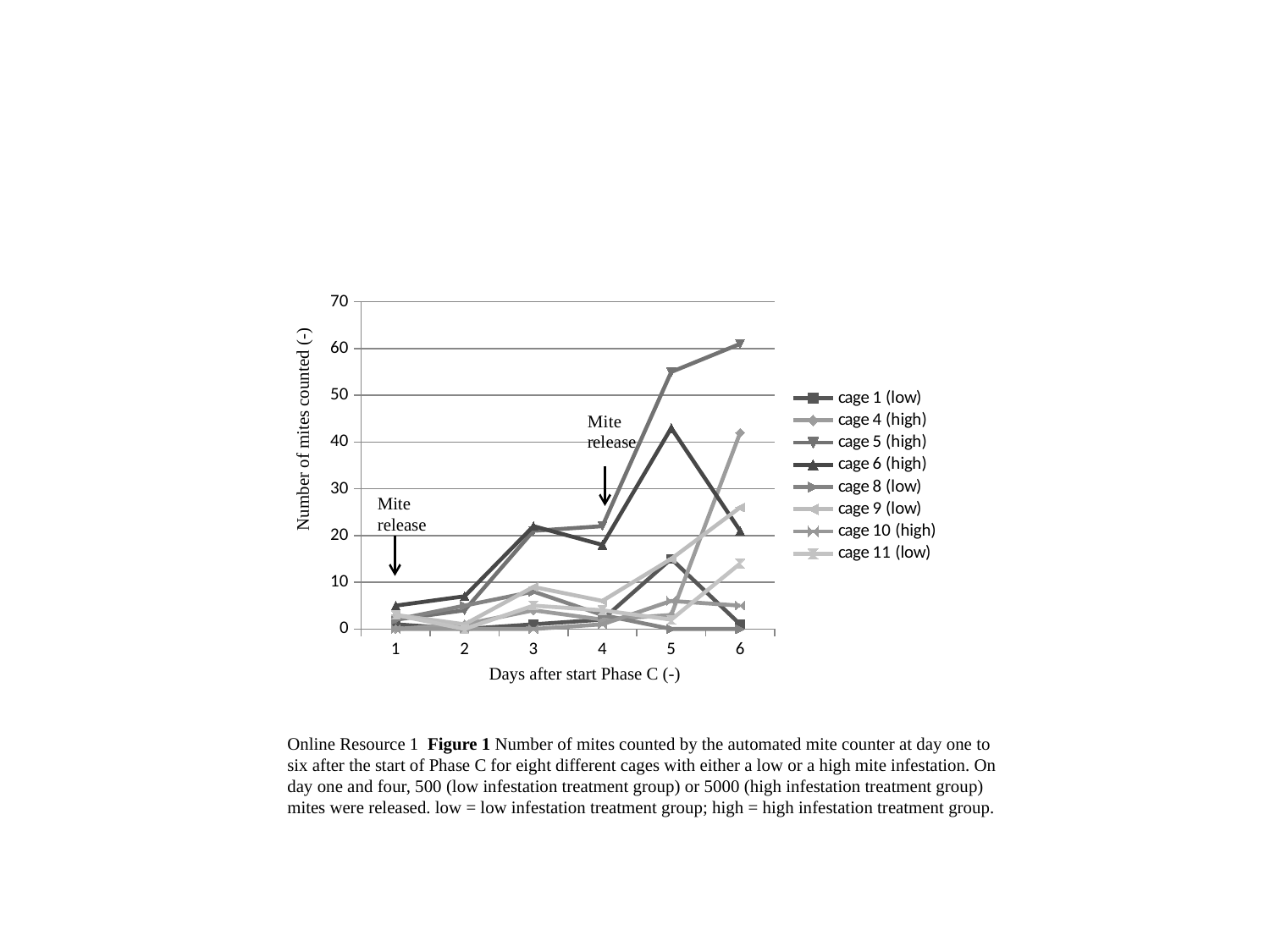

### Chart
| Category | cage 1 (low) | cage 4 (high) | cage 5 (high) | cage 6 (high) | cage 8 (low) | cage 9 (low) | cage 10 (high) | cage 11 (low) |
|---|---|---|---|---|---|---|---|---|Number of mites counted (-)
Mite release
Days after start Phase C (-)
Online Resource 1 Figure 1 Number of mites counted by the automated mite counter at day one to six after the start of Phase C for eight different cages with either a low or a high mite infestation. On day one and four, 500 (low infestation treatment group) or 5000 (high infestation treatment group) mites were released. low = low infestation treatment group; high = high infestation treatment group.
